# Supplementary material for: Atypical calcium regulation of the PKD2-L1 polycystin ion channel
Source: eLife. 2016 Jun 27;5:e13413. doi: 10.7554/eLife.13413 (PMC4922860; doi:10.7554/eLife.13413)
Supplement: Figure 6—source data 1. — Relative permeabilities (Px/PCs) of Na+, K+ and Ca2+ compared to Cs+ were calculated (see Methods) based on the measured reversal potential (N= 4–9, Error ± SEM). DOI: http://dx.doi.org/10.7554/eLife.13413.013 [file elife-13413-fig6-data1.docx]

| **Channel** | **P_X_/P_Cs_ : E_rev_ ± SEM** | | |
| --- | --- | --- | --- |
|  | **Na^+^** | **K^+^** | **Ca^2+^** |
| **Wt** | 3.6 : 6 ± 2 mV | 3.1 : 2 ± 3 mV | 53.7 : 26 ± 2 mV |
| **D525N** | 3.3 : 3 ± 3 mV | 3.7 : 7 ± 2 mV | 8.1 : 16 ± 2 mV |
| **D523N** | 3.6 : 6 ± 2 mV | 3.9 : 8 ± 3 mV | 2.8 : 10 ± 3 mV |
