# Supplementary material for: Atypical calcium regulation of the PKD2-L1 polycystin ion channel
Source: eLife. 2016 Jun 27;5:e13413. doi: 10.7554/eLife.13413 (PMC4922860; doi:10.7554/eLife.13413)
Supplement: Figure 7—source data 1. — Concentration of half current inhibition (IC50) was estimated by fitting the concentration-percent current block relationship using the Hill equation (see Materials and methods). DOI: http://dx.doi.org/10.7554/eLife.13413.016 [file elife-13413-fig7-data1.docx]

| **Chemical or metal** | **Potency of PKD2-L1 current antagonism: IC_50_ (**±**SEM)** | | |
| --- | --- | --- | --- |
|  | **Wt** | **D525N** | **D523N** |
| **Dibucaine** | 31 ± 5 μM | Not tested | 23 ± 3 μM |
| **Cd^2+^** | 25 ± 5 μM | 40 ± 8 μM | No block (< 1 mM) |
| **Zn^2+^** | 156 ± 15 μM | 209 ± 29 μM | No block (< 1 mM) |
| **La^3+^** | 3 ± 4 μM | Not tested | No block (< 1 mM) |
| **Gd^3+^** | 12 ± 5 μM | 12 ± 5 μM | No block (< 1 mM) |
